# Cervicovestibular rehabilitation in adult with mild traumatic brain injury: a randomised controlled trial protocol

**DOI:** 10.1186/s13102-019-0139-3

**Published:** 2019-11-11

**Authors:** Pierre Langevin, Philippe Fait, Pierre Frémont, Jean-Sébastien Roy

**Affiliations:** 1 Clinique Cortex and Physio interactive, 205-1035, avenue Wilfrid-Pelletier, QC Quebec, G1W 0C5 Canada; 2 Department of Rehabilitation, Faculty of Medicine, Université Laval, Pavillon Ferdinand-Vandry, 1050, avenue de la Médecine, bureau 4431, QC Quebec City, G1R 1P5 Canada; 3 Centre for Interdisciplinary Research in Rehabilitation and Social Integration, Quebec Rehabilitation Institute (CIRRIS), 525, Boulevard Wilfrid Hamel, QC Quebec City, G1M 2S8 Canada; 4 Department of Human Kinetics, Université du Québec à Trois-Rivières, 3351, boul. des Forges, QC Trois-Rivières, G8Z 4M3 Canada; 5 Research Center in Neuropsychology and Cognition (CERNEC), Pavillon Marie-Victorin, 90, rue Vincent d’Indy, QC Montreal, H2V 2S9 Canada

**Keywords:** Mild traumatic brain injury, Neck pain, Dizziness, Cervical spine, Vestibular system, Function, Persistent post-concussion symptoms, Gradual activation

## Abstract

**Background:**

Mild traumatic brain injury (mTBI) is an acknowledged public health problem. Up to 25% of adult with mTBI present persistent symptoms. Headache, dizziness, nausea and neck pain are the most commonly reported symptoms and are frequently associated with cervical spine and vestibular impairments. The most recent international consensus statement (2017 Berlin consensus) recommends the addition of an individualized rehabilitation approach for mTBI with persistent symptoms. The addition of an individualized rehabilitation approach including the evaluation and treatment of cervical and vestibular impairments leading to symptoms such as neck pain, headache and dizziness is, however, recommended based only on limited scientific evidence. The benefit of such intervention should therefore be further investigated.

**Objective:**

To compare the addition of a 6-week individualized cervicovestibular rehabilitation program to a conventional approach of gradual sub-threshold physical activation (SPA) alone in adults with persistent headache, neck pain and/or dizziness-related following a mTBI on the severity of symptoms and on other indicators of clinical recovery. We hypothesize that such a program will improve all outcomes faster than a conventional approach (between-group differences at 6-week and 12-week).

**Methods:**

In this single-blind, parallel-group randomized controlled trial, 46 adults with subacute (3 to12 weeks post-injury) persistent mTBI symptoms will be randomly assigned to: 1) a 6-week SPA program or 2) SPA combined with a cervicovestibular rehabilitation program. The cervicovestibular rehabilitation program will include education, cervical spine manual therapy and exercises, vestibular rehabilitation and home exercises. All participants will take part in 4 evaluation sessions (baseline, week 6, 12 and 26) performed by a blinded evaluator. The primary outcome will be the Post-Concussion Symptoms Scale. The secondary outcomes will be time to clearance to return to function, number of recurrent episodes, Global Rating of Change, Numerical Pain Rating Scale, Neck Disability Index, Headache Disability Inventory and Dizziness Handicap Inventory. A 2-way ANOVA and an intention-to-treat analysis will be used.

**Discussion:**

Controlled trials are needed to determine the best rehabilitation approach for mTBI with persistent symptoms such as neck pain, headache and dizziness. This RCT will be crucial to guide future clinical management recommendations.

**Trial registration:**

ClinicalTrials.gov Identifier - NCT03677661, Registered on September, 15th 2018.

## Background

Mild traumatic brain injury (mTBI) is an acknowledged public health problem. It is estimated that between 1.6 to 3.8 million brain injuries occur annually in the United States, with up to 75% classified as mild [1]. The majority of mTBI resolves within 10 to 14 days [2–5]. However, up to 31% of pediatric cases [6] and 25% of adult cases [5, 7] present post concussive syndrome (PCS), which is a persistence of somatic (for example: headache, neck pain, dizziness, nausea, balance dysfunction) [8, 9], cognitive (for example: memory loss and slowed reaction time) [10], and/or psychological (for example: depression and anxiety) [10, 11] symptoms [12]. Among these symptoms, headache and dizziness are the most commonly reported, followed by nausea and neck pain [2, 5, 9]. Many of these PCS symptoms could be explained by injuries to structures near or in the head, other than the brain itself. For example, following a trauma, structures such as the cervical spine, the vestibular ocular system and the temporomandibular joint can be injured. The energy needed to produce an mTBI can be transferred to the neck and produce an injury mechanism similar to the one observed in whiplash associated disorders (WAD). Neck pain, headaches, dizziness and balance dysfunction are common symptoms associated with both mTBI and WAD [5, 13, 14]. Specific interventions aimed at addressing the different underlying cause of these symptoms could lead to improved outcomes. For individuals presenting with PCS, the most recent international consensus statement (2017 Berlin consensus on concussion in sport) [5] recommends the addition of an individualized rehabilitation approach to a sub-threshold physical activation (SPA) strategy. However, this new recommendation is based on limited scientific evidence as well as expert recommendations [5]. Therefore, the effects of adding individualized rehabilitation interventions for the treatment of potential impairments of body function associated with neck pain, headache and dizziness, [5] needs to be evaluated in individuals with mTBI. Several systematic reviews [15–18] have shown that multimodal rehabilitation interventions and vestibular rehabilitation improved function for participants with neck pain, cervicogenic headache, dizziness and balance dysfunction over control. None of the randomized control trials (RCT) studied in these reviews, however, included individuals with mTBI [15–18]. Two RCTs have partially looked at the effects of rehabilitation interventions in some subgroups of mTBI patients. One RCT [19] (*n* = 31) demonstrated that patients with sport-related concussion treated with a standardized combination of vestibular and cervical physiotherapy were quicker to be medically cleared to return to sport than a control group who rested before gradually returning to activities. However, the intervention used in that study did not include an individualized SPA program. Another RCT [20] recruited 41 sport-related concussion patients with dizziness as the main symptom. Participants were quicker to be medically cleared in the rehabilitation treatment targeting dizziness than in the minimal intervention group (subtherapeutic and non-progressive therapeutic techniques). However, due to the multi-factorial nature of mTBI, treatment must be individualized to the patient’s clinical presentation and environment and the outcomes need to encompass all types of symptoms. In that context, there is a need for further RCTs evaluating the effect of an individualized SPA combined with cervicovestibular rehabilitation program (based on the Berlin consensus) on mTBI compared to an individualized SPA alone.

### Objectives and hypothesis

The primary objective of the current RCT is to compare the addition of a 6-week individualized gradual SPA program combined with cervicovestibular rehabilitation program to a gradual SPA program alone in adults with subacute (> than 3 weeks post mTBI) headache, neck pain and /or dizziness-related to mTBI on the severity and impact of symptoms as measured by the Post-Concussion Symptoms Scale (PCSS). The secondary outcomes will be: time for clearance to return to usual activities, number of recurrence episode within 26 weeks after the treatment phase, functional level, intensity of neck pain, headache and dizziness. As SPA is well recognised for the treatment of persistent PCS [5], it supports the choice of using SPA as the comparator intervention in the present study. Our hypothesis is that the individualized SPA combined with cervicovestibular rehabilitation program will improve overall symptoms, time to return to activities as well as function faster and to a greater extent than the conventional approach and between group differences will be observed at week 6 and 12.

## Methods

### Study design

This single-blind, parallel-group RCT will include 8 supervised treatments during a 6-week rehabilitation program and four evaluation sessions over 26 weeks (baseline, week 6 [immediately after the rehabilitation program], week 12 [6 week after the end of the rehabilitation program] and week 26). All participants will take part in the baseline evaluation. After giving informed consent, they will first complete a questionnaire on sociodemographic (age, gender, type of sport or physical activities, number of years playing sport and/or other activities), symptomatology (mechanism of injury, history of previous mTBI, history of dizziness, headache, neck pain and unsteadiness) and comorbidity, as well as self-administered questionnaires that evaluate symptoms and functional limitations, including the PCSS (primary outcome). Once baseline data are collected, participants will be randomly assigned to a control or an experimental group. The control group will receive a 6-week gradual sub-threshold physical activation (SPA) program. The experimental group will receive a gradual SPA program combined with a cervicovestibular rehabilitation program. Between week 6 and week 12, participants will be asked to continue their exercises and follow the advice given at the last meeting with the health professional. Six, 12 and 26 weeks after randomization, all the outcomes will be revaluated (see Table 1). The evaluation sessions will be carried out at the *Centre interdisciplinaire de recherche en réadaptation et en intégration sociale* (CIRRIS) by a research assistant blinded to group assignment, while the interventions will be given at *Clinique Cortex* by experienced physiotherapists, neuropsychologists and kinesiologists. Recruitment began on April 1st 2019 in Quebec City, Canada. See Table 1 for Schedule of enrolment, interventions, and assessments. Ethics approval has been obtained from the Sectorial Rehabilitation and Social Integration Research Ethics Committee of the CIUSSS-CN (#2018–619)*.* The study protocol has been registered on ClinicalTrials.gov [NCT03677661–09/15/2018 - version 1].

**Table 1:**
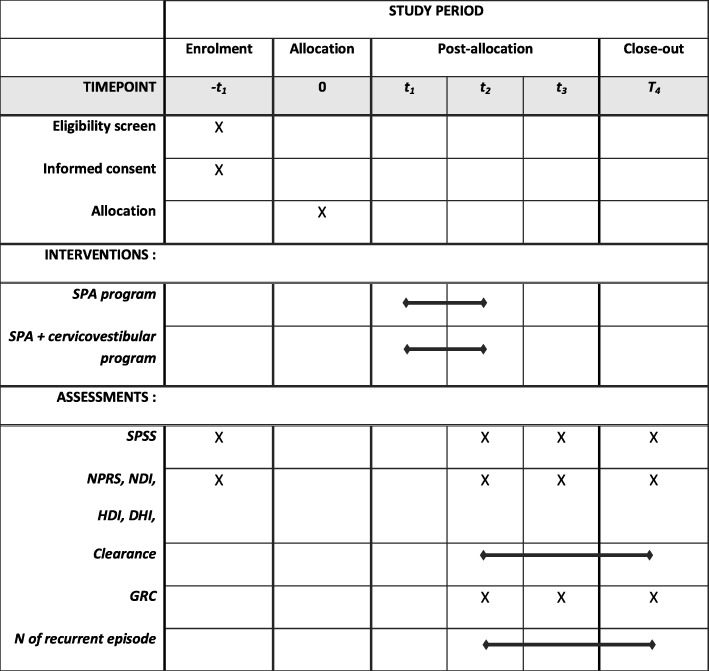
Schedule of enrolment, interventions, and assessments

t_1_ = First intervention session post-allocation, t_2_ = week 6, t_3_ = week 12, t_4_ = week 26. *SPA* Sub-threshold Physical Activity, *SPSS* Post-Concussion Symptoms Scale, *NPRS* Numerical Pain Rating Scale, *NDI* Neck Disability Index, *DHI* Dizziness Handicap Inventory, *HDI* Headache Disability Index, *GRC* global rating of change

### Population

Forty-six adults with a diagnosis of mTBI (based on the definition of McCrory et al.) [5] and symptoms of dizziness, neck pain and/or headaches (reported on the PCSS) [21] lasting for more than 3 weeks will be recruited. Sample size calculation is based on changes evidenced by the PCSS for individuals with mTBI. According to sample size calculation (G*Power 3.1.9.2; α = 0.05, effect size = 0.8, power [1-β] = 0.80, SD = 20.0 PCSS points, MDC = 12.3 PCSS points [22], 10% attrition), a minimum of 23 subjects is needed in each group. The expected attrition (10%) is based on the drop-rate in previous RCT from our group [23–26] and is more than the observed drop-out in similar studies in this population (6% [19] and 2% [20]). Therefore, 46 participants with mTBI will be recruited. Potential participants will be recruited at *Clinique Cortex* (an interdisciplinary concussion clinic specialized in the management of mTBI; 500 new patients with mTBI are evaluated every year), in medical clinics around Quebec City, and through the electronic mailing list of the students and employees at *Université Laval* (> 52,000 individuals).

#### *Inclusion criteria*

- Between 18 and 65 years of age;
- Sustained a mTBI in the past 3 to 12 weeks;
- Having ongoing post-concussion symptoms from the list in the PCSS that started 72 h or less after an impact;
- Having felt at least one or more of the following cognitive symptoms: lost of consciousness for less than 30 minutes following the trauma, feeling slowed down, feeling like in a fog, “don’t feel right”, difficulty concentrating, difficulty remembering and confusion that started 72 h or less after an impact;
- Having abnormalities on one of the following tests: the cervical physical examination (eg, tenderness/spasm/pain on segmental testing, or reduced motion), the vestibular evaluation (eg, Dix halpike test, vestibulo-ocular reflex test, or head thrust test) or the ocular motor evaluation (eg, convergence, smooth visual pursuits, or saccades).

#### *Exclusion criteria*:

- Patients with more than 30 min of loss of consciousness for the current episode;
- Patients with more than 24 h of post-traumatic amnesia;
- Glasgow Coma Scale score lower than 13 at more than 30 min after the injury;
- Patients with radiographic evidence of subdural hemorrhage, epidural hemorrhage, intraparenchymal hemorrhage, and cerebral or cerebellar contusion;
- Post-injury hospitalization for more than 48 h;
- Fracture (head, neck and spine);
- Having a neurological condition, other than the actual mTBI;
- Having a cognitive or behavioural impairment prior to the participation in the study;
- Have had general anesthesia during the three-month period prior to the study;
- Having comorbidities of cardiovascular or respiratory systems.

### Randomisation/blinding

A randomisation list will be generated by an independent research assistant (not involved in data collection) prior to the initiation of the study using a random number generator. Allocation will be concealed in sealed and opaque envelopes that will be sequentially numbered. A blocked randomisation will be used to make sure that two equal groups of 23 participants are obtained. Stratification will be done according to sex to ensure women and men are equally represented in each group as it has been shown that women tend to recover more slowly than man from a mTBI [6, 27]. Given that it is not possible to blind the treating physiotherapist and the participants, a single-blind design will be used as only the evaluator will be blinded. One of the Principal Investigator (PI) will open the randomisation envelope indicating the participant’s assignment and will send the information to the treating therapist. The physiotherapists, neuropsychologists and the kinesiologists will be blinded to the baseline evaluation results. To evaluate the effectiveness of blinding, the evaluator will answer the following question at the week-6 evaluation: *“In your opinion, which intervention did this participant received?”* The possible answers are: 1) SPA program (control group); 2) SPA combined with cervicovestibular rehabilitation program (experimental group); 3) I have no idea. Participants will be unaware of the treatment provided to the participants in the other group. Evaluation will be done in a separate site than the intervention site and participants will be instructed not to reveal or discuss treatment with the evaluator.

### Intervention

All included participants will receive: 1) verbal and written counselling about the current best-practice approach for the treatment of mTBI that consist of gradual cognitive and physical activity that do not result in symptoms exacerbation and 2) individualized recommendations about cognitive and physical activation. A neurocognitive assessment and an exercise tolerance assessment will be used to provide the individualized recommendations regarding gradual cognitive and physical activation. A neuropsychologist will proceed to a clinical neuropsychological assessment of anxiety, attention and executive function using the following tests: Hospital anxiety and depression scale [28], Working memory index and Processing speed index of the WAIS-IV [29], Conners’ Continuous Performance Test 3 [30]. Advice will be provided based on the clinical evaluation results regarding an individualized step-by-step graded exposition to cognitive stimulus guided by symptoms evolution. A follow-up assessment by the neuropsychologist will be held after 6 weeks and advice will be given according to the results of this follow-up assessment. A kinesiologist will also evaluate the symptomatic response to cardio-vascular exertion. The result of this evaluation will be used to provide each participant with a graded physical exercise program aiming for sub-symptoms exacerbation.

#### Control group – gradual SPA program

The subjects in this group will take part in 8 in-clinic cardiovascular exercise sessions in a 6-week period supervised by a kinesiologist (30 to 45 min each session). If the symptoms disappear within the treatment period, the subject will be evaluated for clearance.

#### Experimental group – individualized gradual SPA combined with cervicovestibular rehabilitation program

Two physiotherapists (instead of a kinesiologist for the control group) will supervise the 8 treatment sessions (30 to 45 min each session) according to an individualized care program which will include a cervical and/or vestibular rehabilitation program, as well as the cardiovascular exercise component. The participant will also perform a home cervicovestibular specific-exercise program once a day. The first physiotherapist will be an expert in manual physical therapy. The physiotherapist will evaluate the physical dysfunctions associated to mTBI with a standardized evaluation to build the treatment plan. Cervical and upper cervical range-of-motion and segmental mobility testing using passive physiological intervertebral motion testing for pain reproduction and mobility assessment will be used. Measures of intervertebral mobility assessment have been shown to be reliable at the cranio-vertebral region (Kappa from 0.81 to 0.83) [31]. Segmental mobility testing is reliable at the cervical spine for mobility when combined with pain reproduction (Kappa 0.79 to 0.96) [32]. Muscle strength and coordination will also be assessed using the cranio-cervical flexion test [33]. Temporomandibular function will be assessed for mobility and pain response as described in VonPiekarts and Ludtke trial [34]. Cervical spine proprioception will be evaluated if deemed necessary clinically using the cervical rotation joint position error test [35]. Finally, vestibulo-ocular function using the Vestibular/Ocular Motor Screening (VOMS) [8], head trust, Dix Hallpike and balance using the Balance Error Scoring System (BESS) [36] will be assessed at the first session to create the treatment plan. The physiotherapist will provide cervical and upper cervical mobilisations and manipulation. The mobilisation techniques will be chosen by the physiotherapist according to the results of the examination performed at the beginning of each session with the segmental manual testing procedure described above. The physiotherapists will be allowed to use any of the following manual therapy techniques: cranio-vertebral flexion or extension, rotations at any levels, lateral glides, postero-anterior glides or muscle relaxing technique at the cranio-vertebral region. At the mid and lower cervical spine, infero-medial glides, supero-anterior glides mobilisations or muscle relaxing technique will be used if deemed clinically relevant as described in a previous trial [24]. The therapeutic exercises will consist of range of motion, neuromotor retraining of the neck stabilizers muscles and sensorimotor retraining exercises based on the best current clinical approach for neck pain and cervicogenic headache and according to the impairment specifically found on each patient [14, 16, 37, 38]. The second physiotherapist, will be a vestibulo-ocular expert physiotherapist. He will provide treatment consisting of the canalith repositioning manoeuvre, vestibular adaptation, ocular motor exercises, balance and/or habituation exercises [39, 40]. The vestibular treatment will also be adapted by the treating physiotherapist to the individual participant according to the impairment found at the initial evaluation. Education on concussion and on the neurophysiology underlying their symptoms will be given to participants by the therapist. The number of treatments given by each physiotherapist will vary among participant according to findings at the clinical evaluation but will be limited to a maximum of 8 treatment sessions (30 to 45 min each treatment). Patients in this group will also perform the graded SPAprogram according to the initial kinesiologist recommendation and supervised by the physiotherapist. If the symptoms disappear within the treatment period, the subject will be evaluated for clearance to return to usual activities. Adherence to treatment will be recorded by the treating therapists and a logbook will be completed by the participants each week to record home exercises performed. All participants will be advised to avoid concomitant interventions. If a concomitant intervention is used, it will be recorded by the treating therapist. Table 2 describes every intervention step that both groups will achieve.

**Table 2:** Description of the intervention

Control – SPA program	All subjects: Neuropsychologist and kinesiologist advice	Control: Cardiovascular program only - 8 sessions	All subjects: Follow-up by the neuropsychologist for final advice
Intervention - SPA + cervicovestibular program		Intervention: Cervicovestibular physiotherapy and cardiovascular program - 8 sessions	

### The primary outcome measure

#### *Post-Concussion Symptoms Scale (PCSS)*

The severity and impact of symptoms will be measured by a self-reported scale, the PCSS [41]. This scale is a list of 22 symptoms for which participant rate each symptom for severity on a 0 (none) to 6 (severe) numerical scale. The maximum possible score is 132 (22 × 6 = 132). This valid and reliable scale has a minimal detectable change (90% confidence interval) of 12.3 PCSS points [22]. Normative values have been established [41]. The symptoms list can be divided in four main sub-groups (physical, cognitive, emotional and sleep disorders) and analysed accordingly [5].

### Secondary outcome measures

The secondary outcome measures will include: Clearance to return to pre-injury function without restriction, Numerical Pain Rating Scale, Neck Disability Index, Dizziness Handicap Inventory, Headache Disability Index, number of recurrent episodes and global rating of change.

#### Clearance to return to function

The number of days between the initial evaluation, the date of the trauma and the full clearance to return to function (work, study or physical activity/sport) will be measured. The clearance to return to function will be determined by the treating therapist and the neuropsychologist. The treating therapist (physiotherapist or kinesiologist according to which group the patient will be allocated to) will use the stepwise progression from the 5th International Consensus Statement on Sport Concussion [5]. As patients achieve the last step of this progression, indicating that the patient can safely return to play, the neuropsychologist and the kinesiologist will confirm this decision with a structured interview (neuropsychologist) and an ergocycle aerobic test (kinesiologist). The clearance will be determined by the day for which 1) the symptoms will have return to usual level 2) the neurological, cervical spine and vestibular impairments found at the beginning of the study will be considered in the normal range by the treating therapist 3) the subject returned to his normal level of all functional activities.

#### Numerical pain rating scale (NPRS)

The level of neck pain and headache will be captured separately with NPRS. Using an 11-point scale, ranging from 0 (no pain) to 10 (worst pain imaginable), participants will be asked to answer the following question: “On a scale of 0 to 10, where 0 corresponds to no pain and 10 to the worst imaginable pain, evaluate the intensity of your neck pain at this moment”. The same question will be asked for the headache. The NPRS is moderately reliable (Intraclass Correlation Coefficient [ICC] = 0.76) and has a clinically important difference of 13% [42].

#### Neck disability index (NDI)

The NDI is a 10-item questionnaire that measures a patient’s self-reported neck pain related disability. Questions include pain and activities of daily living. The questions are measured on a six-point scale from 0 (no disability) to 5 (full disability). The numeric response for each item is summed for a total score ranging from 0 to 50. The reliability (ICC: 0.73 to 0.98), construct validity, and responsiveness to change have all been demonstrated in various populations [43]. The validated French version NDI will be used [44].

#### Headache disability inventory (HDI)

The HDI is a 25-item questionnaire measuring the disability related to patient reported headache. Questions include activities of daily living and perceived disability as measured with an ordinal scale (yes (4 points), sometimes (2 points), no (0 point)). After adding every numerical score, the total score is on 100 for which 0 means no disability and 100 complete disability. The test-retest reliability (r = 0.79 to 0.83) and the minimal detectable change (16 points) are known [45].

#### Dizziness handicap inventory (DHI)

The DHI [46] is a 25-items questionnaire that identifies the degree of perceived difficulty a patient may experience as a result of dizziness or unsteadiness. The items are sub grouped into three content domains representing functional, emotional, and physical aspects of dizziness and unsteadiness [46]. The questionnaire demonstrated high Test-retest reliability (r = 0.92 to 0.97) and internal consistency (α = 0.72 to 0.89) [47].

#### Recurrent episodes

The number of recurrence episodes was calculated as the number of episodes of symptoms with a duration of at least 48 h following a trauma during the 26 weeks of the study.

#### Global rating of change

GRC questions are designed to quantify a patient’s perceived improvement or deterioration over time. Using a 15-point GRC scale, ranging from − 7 (a very great deal worse) to 0 (about the same) to + 7 (a very great deal better), participants will be asked to answer the following question: “Overall, has there been any change in your condition since the initial evaluation? Please indicate if there has been any change in your condition by choosing one of the following options [48].” The validity, reliability (ICC = 0.90) and responsiveness of GRC scales have been established [49].

### Statistical analyses

Descriptive statistics will be used for all outcome measures at each measurement time to summarize results. Baseline demographic data will be compared (independent *t*-test and Chi-squared tests) to establish the comparability of groups. All data will be tested to check the distributional assumptions for the inferential statistical analyses. An intention-to-treat analysis will be used in which all participants will be analysed in the group to which they were originally assigned. Per protocol analysis will also be performed. All dropouts and the reason for dropping out of the study will be reported. Any harm or unintended effects during the programs will be recorded. A 2-way ANOVA (2 Groups [Group 1 or 2] × 4 Time [week 0, 6, 12, 26]) will be used to analyse the effects of the rehabilitation programs on the primary outcome and on most of the secondary outcomes (SPSS 22, proc. GENLIN). We expect no group effects, as the groups should be equal at baseline. A time effect should be observed, as both groups should improve given they will both receive an interventions. Finally, we expect a significant Time x Group interaction since the groups should react differently over time, ***with a faster recovery for the Experimental group mainly seen at week 6 and 12***. This will be statistically detailed with post-hoc tests (Bonferroni correction). An independent t-test will be used to analyse the effects of the rehabilitation programs on clearance to return to function.

## Discussion

This project may have a direct impact on clinical practice in the management of mTBI. Given that the number of reported mTBI and the awareness of the general population about this health condition are growing; and given that prolonged symptoms duration in PCS is often multifactorial and complex, this RCT will help to better define and understand the role of cervical spine and vestibular impairments underlying the symptoms reported in this population. This RCT will also establish the efficacy of individualized cervical and vestibular rehabilitation approaches when added to a cardiovascular rehabilitation program and cognitive behavioral strategies compared to cardiovascular rehabilitation program and cognitive behavioral strategy alone. Physiotherapists have been involved in the functional treatment of mTBI for a long time. However, the treatment of cervical spine and vestibular impairments in mTBI remains at is infancy in the rehabilitation history of mTBI management. It has recently been added to clinical recommendations in 2017 but there is a need to further document the potential of these interventions with well-designed RCTs. This project will help to build knowledge on individualized multidisciplinary strategies to address persistent symptoms following an mTBI.

## Data Availability

The dataset used and/or analyzed during the current study is available from the corresponding author on request.

## Abbreviations

ANOVA: Analysis of variance
BESS: Balance Error Scoring System
CIRRIS: Center for Interdisciplinary Research in Rehabilitation and Social Integration
DHI: Dizziness Handicap Inventory
GRC: Global rating of Change
HDI: Headache Disability Index
ICC: Intraclass Correlation Coefficient
mTBI: Mild Traumatic Brain Injury
NDI: Neck Disability Index
NPRS: Numerical Pain Rating Scale
PCSS: Post-Concussion Symptoms Scale
RCT: randomised clinical trials
VOMS: Vestibular/Ocular Motor Screening
WAD: Whiplash Associated Disorder
